# The Medical Inspection of Scholars

**Published:** 1906-07-28

**Authors:** 


### The Hospital

A Journal of

The Medical Sciences and Hospital Administration.

Vol. XL.—No. 1,036. SATURDAY, JULY 28, 1906.

### The Medical Inspection of Scholars.

The debate in the House of Commons, on Monday week, on the medical inspection of the children attending elementary or other State-aided schools was so far satisfactory that it leaves both political parties fully committed to the importance, or even to the necessity, of such a proceeding; and hence, whatever may be the final shape in which the present Bill is carried to the House of Lords, or in which it may issue from that assembly, and whether or not it may be fated to receive the Royal assent, it is at least certain that powers to conduct medical inspections will either form part of it, or will be incorporated, almost as non-contentious matter, in any other by which it may be succeeded. That principle of inspection is the simple one that the Government has no moral, and should have no legal right, to inflict injury upon children under the guise of benefit. The State, in rendering education, or rather schooling, compulsory, is bound to secure that it shall at least be harmless; and there is already no lack of evidence to show that, in cases of underfeeding, of constitutional debility, and of certain affections of the eyes, this obligation has not hitherto been adequately fulfilled. Every medical man in private practice is from time to time consulted by parents concerning modifications of common methods of instruction which the personal needs of individual children may render necessary; and it is only too common an experience that such consultations are often unduly delayed. A well-known writer on ophthalmology has made the general statement that he had scarcely ever, if ever, been consulted about a child with defective sight who had not been bullied or punished for supposed carelessness or stupidity before the defect had been either discovered or suspected. If this were the case among families in easy circumstances, how much more is it likely to be so among the poor, unless in cases in which the intelligence of the teacher may serve to correct the ignorance of the parent; and the poor, although they may rightly be called upon to supply their children with necessaries to the extent of their powers, cannot reasonably be expected to take them to specialists. To this extent the State, which enforces the teaching, is called upon to protect the child; and the same principle should apply to cases of defective hearing, or of adenoids, in which, although the child itself would not be likely to sustain injury from the teaching, its power of deriving benefit would be so limited that the expense incurred upon its account would in great measure be thrown away. In such conditions, it is evident, the State may fairly be called upon to protect the ratepayers.

In addition to these sources of demand for medical supervision, there is the further consideration that the systematic medical inspection of children of school age will supply, in the course of a few years, a great gap in the vital statistics of the nation, and will bring into the region of fact many assertions which at present rest almost entirely upon conjecture. The proposal of systematic inspection is one that must be welcomed by all classes of the community, and it is manifestly urgent that prompt and proper provision should be made for it. It is only when we consider the nature of this provision that differences of opinion seem likely to arise, and that the suggestions of the penny wisdom which is associated with pound foolishness begin to assume some prominence.

Sir Gilbert Parker, in generally supporting Mr. Tennant's proposal that "it shall be the duty of every local education authority to make arrangements, in accordance with a scheme to be made by the Board of Education, for attending to the health and physical condition of the children educated in public elementary schools," unfortunately committed himself to the statement that "it would be possible to get the medical officers of health to carry out this work at no very great expense to the public." We can neither agree with this opinion nor feel anything but regret that a policy of cheeseparing should be permitted to show itself in relation to this question. Medical officers of health are of two classes, those who are debarred from private practice and limited to sanitary functions, and those in charge of small areas, who are permitted to practise, and who, we trust it may be said without offence, are frequently in the employment of local authorities not particularly solicitous about the discharge of their duties. Gentlemen of the former class, who may perhaps have never been in practice, are not likely to be the most effective examiners of individual children; and gentlemen of the latter class would not always carry sufficient weight with the school managers of their own districts. There could be no objection to committing a general superintendence of the inspection to the medical officer of health for the county, or for a populous borough, who might do much to harmonise the work of individual inspectors; but this work should, we think, be mainly committed to practitioners of experience, and, in the case of girls, to lady practitioners whenever circumstances would so permit. It might even be desirable to have a visiting inspecting staff, which should be attached to the Board of Education, and be entirely independent of locality.

